# Parallel and Scalable Short-Read Alignment on Multi-Core Clusters Using UPC++

**DOI:** 10.1371/journal.pone.0145490

**Published:** 2016-01-05

**Authors:** Jorge González-Domínguez, Yongchao Liu, Bertil Schmidt

**Affiliations:** 1 Parallel and Distributed Architectures Group, Johannes Gutenberg University Mainz, Mainz, Germany; 2 School of Computational Science & Engineering, Georgia Institute of Technology, Atlanta, Georgia, United States of America; Oak Ridge National Lab, UNITED STATES

## Abstract

The growth of next-generation sequencing (NGS) datasets poses a challenge to the alignment of reads to reference genomes in terms of alignment quality and execution speed. Some available aligners have been shown to obtain high quality mappings at the expense of long execution times. Finding fast yet accurate software solutions is of high importance to research, since availability and size of NGS datasets continue to increase. In this work we present an efficient parallelization approach for NGS short-read alignment on multi-core clusters. Our approach takes advantage of a distributed shared memory programming model based on the new UPC++ language. Experimental results using the CUSHAW3 aligner show that our implementation based on dynamic scheduling obtains good scalability on multi-core clusters. Through our evaluation, we are able to complete the single-end and paired-end alignments of 246 million reads of length 150 base-pairs in 11.54 and 16.64 minutes, respectively, using 32 nodes with four AMD Opteron 6272 16-core CPUs per node. In contrast, the multi-threaded original tool needs 2.77 and 5.54 hours to perform the same alignments on the 64 cores of one node. The source code of our parallel implementation is publicly available at the CUSHAW3 homepage (http://cushaw3.sourceforge.net).

## Introduction

The application of next-generation sequencing (NGS) technologies has led to an explosion of short-read sequence datasets. The alignment of produced sequences to a given reference genome, i.e. short-read alignment (SRA), is one of the most important basic operations required for further downstream analysis. Continuous improvements of NGS technologies have led to a steady increase in throughput by producing more reads as well as increasing average read length. However, longer reads often come at the expense of higher sequencing error rates. A variety of aligners, such as GASSST [1], Bowtie2 [2], GEM [3], SeqAlto [4], BWA-MEM [5] and CUSHAW3 [6], have been proposed in order to deal with these features. All of them are based on the *seed-and-extend* approach, although using different seeding policies. This approach maps a given read by first identifying seeds on the genome using efficient indexing data structures. Seeds are then extended (e.g., by using fast versions of dynamic programming based alignment algorithms) in order to verify if a seed can actually be extended to a full alignment. Note that the time needed for the alignment of each read can vary as it depends on the number of associated seeds.

Even though the seed-and-extend approach is relatively efficient, associated SRA runtimes can still be high since NGS datasets can contain hundreds of millions or even billions of reads. Parallelization can be used to reduce these runtimes. Many aligners take advantage of the capabilies of common multi-core CPUs by using multi-threading and SIMD vectorization. Furthermore, the usage of modern accelerator hardware for SRA has attracted research interest. The most popular accelerator architecture for SRA are GPUs with some examples of GPU-based tools being CUSHAW [7], BarraCUDA [8], SOAP3-dp [9], and nvBowtie [10]. Other examples include FPGA and Xeon Phi implementations such as [11] and [12].

So far, not many efforts have been made to develop tools able to exploit the characteristics of compute clusters. pMap [13] and pBWA [14] use MPI to distribute sequence reads among the processes and align the assigned reads on each process. While pBWA is limited to a certain version of the BWA aligner, pMap is portable enough to be able to work with a number of different aligners. The current publicly available version of pMap provides support for some popular aligners. Moreover, the source code can be modified in the case that a user wants to work with a new aligner. pMap and pBWA both suffer from two major problems that limit their scalability. First, the overhead of their initial splitting is significant, especially when increasing the number of processes. Moreover, they apply a block distribution that assigns the same number of reads to each MPI process. As the time to align each read in the seed-and-extend approach can vary, a simple block distribution cannot achieve good load balancing. Recently, Hadoop-based tools SEAL [15] and BigBWA [16] have also been introduced to parallelize SRA for cloud computing. Unfortunately, they achieve similar speedups to pMap when using the same underlying aligner and running on a cluster [16].

In this paper, we describe a parallel implementation of a short-read aligner for multi-core clusters with improved scalability compared to pMap. CUSHAW3 [6] has been chosen because of its high alignment quality (which made it suitable for recent studies such as [17]) but high runtime (even using the multi-threaded version). The results in [6] show that CUSHAW3 consistently outperforms BWA-MEM, Bowtie2 and GEM in terms of single- and paired-end quality alignment. CUSHAW3 has also proved to perform highly competitive on several datasets of the GCAT benchmark (http://www.bioplanet.com/gcat). However, it is on average 2.58 and 4.93 times slower than Bowtie2 and BWA-MEM, respectively, on a workstation with two hex-core Intel Xeon X5650 processors. Nevertheless, our parallel approach can be adapted to other seed-and-extend based SRA tools, since the underlying algorithm for aligning one read (or read-pair) does not need to be modified.

## Background

### UPC++ Parallel Programming Language

Our parallel implementation overcomes the scalability issues of pMap thanks to an efficient use of UPC++ [18], an extension of C++ for parallel computing which has evolved from Unified Parallel C (UPC) [19]. PGAS (Partitioned Global Address Space) languages (such as UPC, Co-Array Fortran [20] or Titanium [21]) are often easier to use than message passing counterparts [22, 23] and can also obtain better performance than them thanks to efficient one-sided communication [24–26]. UPC++ combines these advantages of the PGAS model and object oriented programming. Both UPC and UPC++ have recently been used for the parallelization of bioinformatics applications [27–29].

Among them, merAligner [29] is a parallel UPC short-read aligner for distributed-memory architectures which obtains good scalability on multi-core clusters. merAligner optimizes the distribution of the reference genome index in case that it is too large to fit in one node. However, the goal of our work is the parallelization of the type of aligners mentioned before, that work with index data structures that typically fit in the main memory of one node. For instance, for a human reference genome the memory consumption of CUSHAW3 is only around 3 GB.

The execution model of UPC++ is single program multiple data (SPMD). As this language is able to work on both shared-memory and distributed-memory systems, each independent execution unit (from now, UPC++ process) can be implemented as an OS process or a POSIX thread (Pthread). UPC++ takes advantage of C++ language features, such as templates, object-oriented design, operator overloading, and lambda functions (in C++ 11) to provide advanced PGAS features.

**Fig 1:**
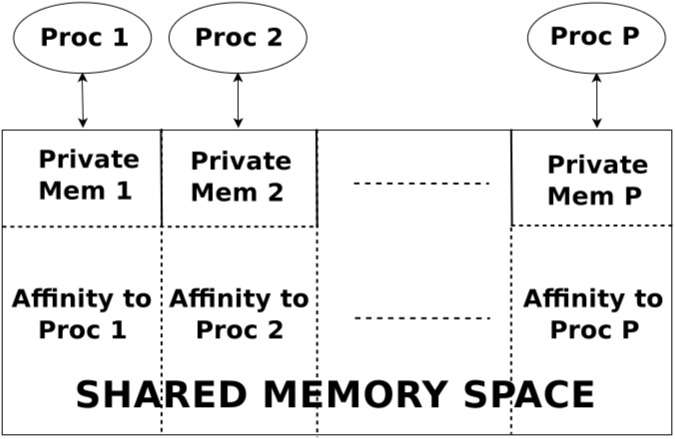
UPC++ memory model.

As all PGAS languages, UPC++ exposes a global shared address space to the user which is logically divided among processes, so each process is associated or presents affinity to a different part of the shared memory. Moreover, UPC++ also provides a private memory space per process for local computations, as shown in Fig 1. Therefore, each process has access to both its private memory and the whole global memory space (even the parts that do not present affinity to it) with read/write functions. This memory specification combines the advantages of both shared and distributed programming models. On one hand, the global shared memory space facilitates the development of parallel codes, allowing all processes to directly read and write remote data without explicitly notifying the owner. On the other hand, performance can be increased by taking data affinity into account. Typically, accesses to remote data are more expensive than the accesses to local data (i.e. accesses to private memory and to shared memory with affinity to the process).

UPC++ provides different mechanisms to synchronize the computation of the processes, such as barriers, locks or asynchronous functions. Among them, our implementation makes use of a shared lock to protect the access of the processes to certain positions of the shared memory. A lock is stored in shared memory and thus it can be accessed by all processes. The basic concept of a lock is that only one process can own it at any given time. Therefore, even if several processes try to access the lock only one will be successful. No other process can access that lock until the owning process unlocks it.

### CUSHAW3

CUSHAW3 is an open-source, multi-threaded, sensitive and accurate short-read aligner supporting both base-space and color-space sequences. This tool is based on the well-known seed-and-extend heuristic, but introduces hybrid seeding approaches to improve the quality of both single-end and paired-end alignments. Concretely, it incorporates maximal exact match (MEM), exact-match *k*-mer and variable-length seeds.

**Fig 2:**
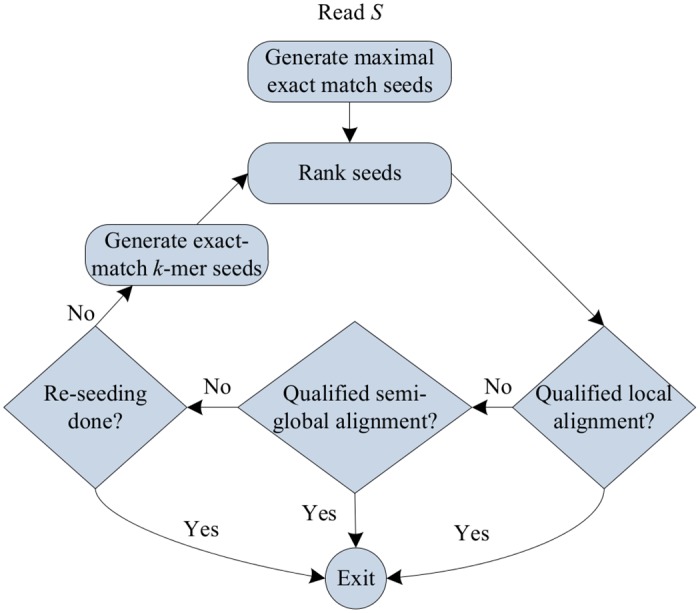
Workflow of CUSHAW3 for the single-end alignment of each read.

Fig 2 illustrates the pipeline of CUSHAW3 to perform the single-end alignment of one read. It starts by generating the MEM seeds of the read and, for each seed, determines a potential mapping region on the reference genome by calculating the optimal local alignment score. All seeds are subsequently ranked in terms of this optimal local alignment score. The next step consists in using dynamic programming from the highest ranked seeds to identify the optimal local alignment of the read on the genome. CUSHAW3 considers this alignment as qualified if it satisfies certain previously specified constraints. Otherwise, CUSHAW3 tries to rescue the read using a semi-global alignment approach. Again, the semi-global alignment is set as qualified if it fulfills certain conditions. Otherwise, it means that the true alignment is not implied by any of the MEM seeds. In this case, CUSHAW3 applies a second rescue by generating the exact-match *k*-mer seeds and performing the same procedure as for MEM seeds. Finally, if the alignments generated by these new seeds also fail to fulfill all the conditions, the read is reported as unaligned.

**Fig 3:**
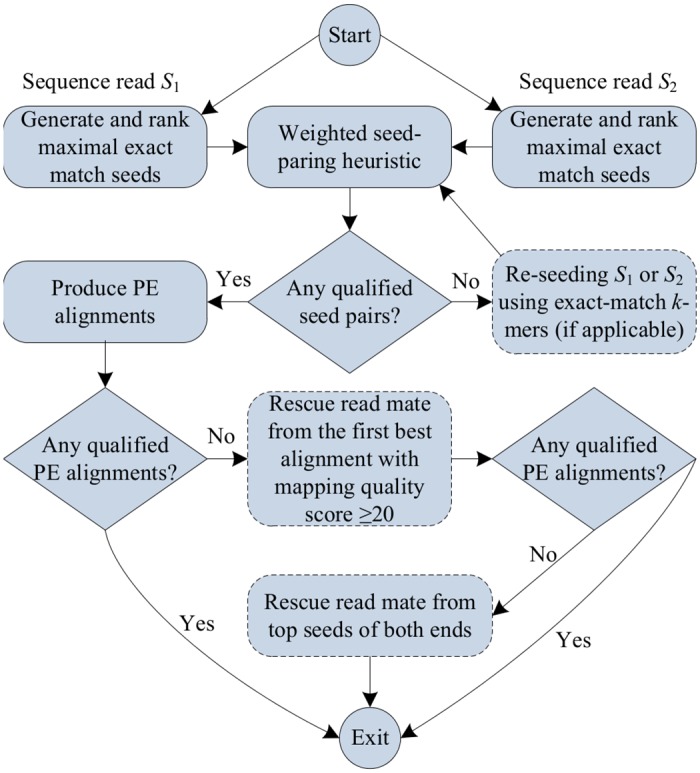
Workflow of CUSHAW3 for the paired-end alignment of each couple of reads.

In comparison to single-end alignment, the information contained in paired-end alignment usually allows to obtain more accurate results thanks to applying techniques such as weighted seed-pairing heuristics, pair ranking and read mate rescuing. CUSHAW3 obtains paired-end alignments with high quality by integrating the hybrid seeding and these three techniques, at the cost of a high computational complexity. Fig 3 shows the workflow of CUSHAW3 for paired-end alignment. It starts by generating and ranking the MEM seeds of the two reads, following the same procedure as in the single-end counterpart. The second step consists of pairing seeds with a weighted seed-pairing heuristic that only takes into account top ranked seeds. Then, CUSHAW3 analyzes if some seed pairs fulfill certain conditions and thus they are qualified. If no seed pair is qualified, the tool repeats the previous step but looking for exact-match *k*-mer seeds. Once at least one seed pair is qualified, CUSHAW3 continues its workload by calculating the real alignments of both ends and identifying these alignments as qualified depending on if they satisfy the insert-size constraint. Finally, read mate rescuing is applied if all alignments are not qualified.

We refer to [6] to find more information about the methodology and the accuracy of CUSHAW3, as well as its comparison to other aligners.

## Methods

The aim of our parallel implementation is to accelerate the SRA performed by CUSHAW3 but preserving the high quality of its results. Therefore, we have not modified the C++ methods in the original source code to align one read or read-pair to a reference genome. The computation starts with all processes reading the genome and saving it into their private memories. Then, the processes apply several times the CUSHAW3 method to align their assigned reads to their copy of the genome.

In the original multi-threaded CUSHAW3 code only one stream is created to access each input file. All threads share this stream so the last accessed position in the file is updated for all threads every time one sequence is read. A mutex variable is created for synchronizing these accesses and thus guaranteeing that each sequence is aligned only once. Unfortunately, this synchronization means that only one thread can access the file at a time. The threads read one sequence from the input file and then call the mentioned C++ method to align it. The main problem of this approach is that the time that threads need to wait in the synchronization step (while other threads access the file) might be long, especially on platforms with a significant number of cores. Directly exporting this approach to UPC++ could be possible with the use of only one shared lock for all the processes. However, the performance would not be satisfactory as we could have thousands of processes idle during the whole time that one process accesses one line of the file. In our implementation we take advantage of the UPC++ capabilities to read the same file from different processes employing different streams. We indicate to each process which reads should be aligned by itself and which ones should be skipped since they are aligned by other processes. With this information all the reads from the input files can be aligned in parallel.

Furthermore, the UPC++ framework included in the parallel implementation works with blocks of reads, i.e., the processes take *block*_*size* reads every time they access the file and then they call the C++ SRA method *block*_*size* times. Therefore, the number of accesses to the file is reduced. The *block*_*size* value can be specified by the user in a configuration file at compile time.

Finally, our UPC++ implementation is compatible with the multi-threaded CUSHAW3 version. In a system with *N* compute nodes containing *C* cores each, we can select only *N* UPC++ processes (one per node) and *C* threads aligning each block, or other combinations up to using *N* × *C* single-threaded processes.

### On-Demand Distribution

An analysis of the CUSHAW3 workflow (see Figs 2 and 3) indicates that the time needed to map each read can vary. In general, the runtime is mainly subject to the following three factors: (*i*) the number of seeds generated, (*ii*) whether the local and semi-global alignments are good enough, and (*iii*) the non-uniform memory access (NUMA) architecture in modern multi-CPU computers. Moreover, for paired-end alignment, the runtime is also effected by the possibility of applying a read mate rescue. Therefore, a static distribution of reads to processes, with the same number of reads for each process, might lead to unbalanced workloads. Our approach applies an on-demand distribution, where the blocks are not initially assigned to the threads, in an attempt to balance the workload. A similar approach was already presented in [30] but only for shared-memory NUMA platforms using Cilk. The procedure for single-end alignment consists of the following steps:

1. All processes load the reference genome into their local memory.
2. Process *i* accesses the input file with its stream and saves into a buffer the information of the reads of the block *i*. To do this it has to skip the information of the previous *i* − 1 blocks of reads.
3. Each process aligns the reads stored in the buffer with *block*_*size* calls to the CUSHAW3 alignment functions. The mappings are written to an intermediate output file associated to the process. Each process has one intermediate file and can work with several threads.
4. As soon as a process finishes the alignment of the block, it checks for the first bock that has not already been aligned by any process.
5. The process skips the reads contained in the blocks that have already been aligned by any process and stores in the buffer the information of the next block (the next *block*_*size* reads).
6. The procedure is repeated from Step 3 until all the reads of the input file have been aligned.
7. The mapping information of the intermediate files is gathered into the output file.

An integer variable stored in shared memory is used to save the number of the first block of sequences that has not been aligned yet by any process. This variable can be accessed and modified by all processes. At the end of Step 5 its value is incremented. The accesses and modification of this shared variable must be synchronized with a lock, in order to avoid race conditions and guarantee that each block is only aligned by one process. Although this synchronization leads to certain performance overhead, its impact is limited because the lock is only held by one process for the short time needed to read and modify the variable. This is a better idea than just adapting the multi-threaded CUSHAW3 approach, in which the processes hold the mutex all the time while they read the input file. Paired-end alignment works in a similar way to singled-end alignment, except that each process reads from two files at the same time.

Note that the shared memory and the locks available in UPC++ allow us to perform this on-demand distribution in an efficient way, where only those processes that finish the alignment of one block at the same time need to be synchronized. In contrast, a conventional message passing approach like MPI would force us to broadcast the index of the next block to be aligned, each time one process computes one block, with its corresponding synchronization among all processes. Nevertheless, we found a problem in the implementation of locks in the current UPC++ version. If Process 0 accesses the lock, it does not release it until the end of the computation, even if we explicitly indicate the release of the lock in the code with the proper directive for unlocking. Therefore, we could not allow Process 0 to align reads. In our implementation, Process 0 is only dedicated to gather the results in the output file once any process *i* (*i* ∈ [1, *P*)) finishes the alignment. Each time one process finishes the alignment, it updates a shared variable to indicate that one intermediate file has been finished. Process 0 continuously polls these variables to check whether other processes have output information and, if this is the case, copies the information to the output file and deletes the corresponding intermediate file. The accesses to these variables do not need to be synchronized as only one process reads them. Algorithm 1 illustrates the pseudocode of our UPC++ on-demand distribution for single-end alignment. As mentioned before, the paired-end counterpart is similar but accesses two input files.

**Algorithm 1** Pseudocode of the UPC++ single-end alignment parallelization

INPUT: FILE stream *F*; intermediate FILE streams *interF*[*P*]; number of processes *P*; rank of the process *MYPROC*; number of threads per process *T*;

SHARED: lock *l*; *nextBlock*; *end*[*P*]

1: *nextBlock* = *P* − 1    // the next block that must be computed

2: *newInfo*[*MYPROC*] = 0      // MYPROC has not finished

3: **if** *MYPROC* = 0 **then**

4:  *procsEnd* = 0    // no process has reached the end

5:  **while** *procsEnd* < *P* − 1 **do**

6:   **for** *i* = 1‥*P* − 1 **do**

7:    **if** *end*[*i*] = 1 **then**      // process i has finished

8:     Copy the information stored in *interF*[*i*] to *F*

9:     Delete *interF*[*i*]

10:     *end*[*i*] = 0

11:     *procsEnd* = *procsEnd* + 1

12:    **end if**

13:   **end for**

14:  **end while**

15: **else**    // processes different than 0

16:  load the genome into memory

17:  *myBlock* = *MYPROC* − 1    // the first block to align

18:  **while** Exits *myBlock* in *F* **do**

19:   align *myBlock* using *T* threads

20:   write the results in *interF*[*MYPROC*]

21:   lock *l*    // start of the synchronized accesses

22:   *myBlock* = *nextBlock*

23:   *nextBlock* = *nextBlock* + 1

24:   unlock *l*    // end of the synchronized accesses

25:  **end while**

26:  *end*[*MYPROC*] = 1

27: **end if**

The advantage of our on-demand distribution is that the workload adapts to the characteristics of the input file. One process computes more blocks if the reads that are assigned to it are aligned fast. Finally, note that the choice of *block*_*size* has an impact on the performance. Increasing *block*_*size* decreases the number of blocks and thereby reduces the amount of synchronization among processes. On the contrary, a larger *block*_*size* also restricts the adaptability of the on-demand approach and could lead to a more unbalanced workload.

## Results and Discussion

32 nodes of the MOGON cluster, installed at the Johannes Gutenberg University Mainz, are used for evaluating the scalability of our parallel SRA tool. Each node contains four 16-core AMD Opteron 6272 processors (i.e., 64 cores at 2.10 GHz within each node). A private L1 cache of 16 KB is available for each core, while the 2 MB L2 and 8 MB L3 caches are shared among two and eight cores, respectively. Nodes provide 128 GB of memory and are connected through a QDR InfiniBand network. UPC++ runs over GCC v4.8.1 and OpenMPI v1.6.5.

We have analyzed the scalability of our UPC++ implementation by aligning four Illumina short-read datasets (see Table 1) to the human genome hg38. In Table 1, all datasets are publicly available and named after their accession numbers in the NCBI sequence read archive. Remark that in this experimental evaluation we only focus on analyzing the performance in terms of speed. We have tested that the output alignments provided by the parallel implementation are the same as for the original CUSHAW3, so we do not need to check again the accuracy of the results (see [6, 31] for more information). The only difference is that the order of the alignments in the output file can be different, which does not influence alignment quality. Furthermore, although CUSHAW3 and our UPC++ version also support color-space reads, we have focused our evaluation on the alignment of more common base-space datasets. Nevertheless, we expect to achieve similar scalability on color-space datasets as the UPC++ parallelization does not modify the method to align each read. The C++ method for base- or color-space alignment can be seen as a black box. Thus, the features of our parallel implementation are directly exported to color-space experiments.

**Table 1:** Characteristics of the Illumina datasets used in the tests.

Name	Number of reads	Length of reads
SRR034939	36,200,062	100
SRR211279	50,937,050	100
SRR091634	328,621,238	100
SRR926245	246,839,706	150

A preliminary performance evaluation of the UPC++ implementation was performed by varying *block*_*size* from 500 to 50,000 reads. The best performance depends not only on the number of processes but also on the characteristics of the input dataset. Thus, we cannot guarantee that a certain value of *block*_*size* will always be the best choice. In this regard, for all the experiments, we have set *block*_*size* to 5,000 for the on-demand approach, as this setting is observed to obtain reasonable performance in all cases through our evaluations.

### Single-End Alignment

**Fig 4:**
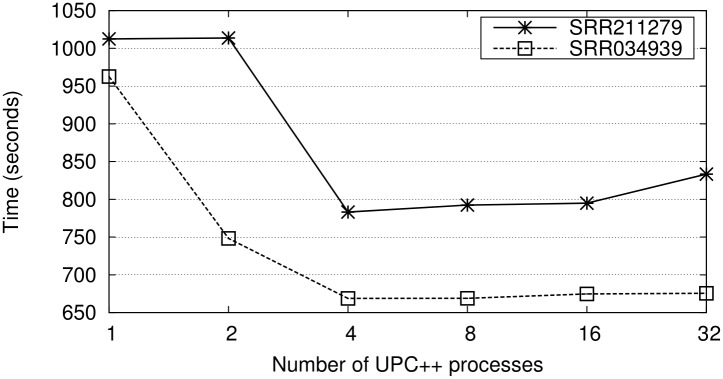
Runtimes (seconds) of the single-end alignment using all the 64 cores of one node and varying the combinations of proccesses/threads (from one process with 64 threads to 32 processes with two threads each).

Each node has multiple CPU cores, therefore tuning the number of processes per node and the number of threads per process is important for both runtime and memory overhead. Hence, we have used the two smallest datasets to analyze the best combination of UPC++ processes/threads for the alignment within each node. Fig 4 illustrates the runtime for the single-end alignment for all possible combinations. Note that using several processes per node increases the memory requirements, as each process saves its own copy of the genome. In our case the nodes have enough memory (128 GB) to store up to 32 copies of the genome. Therefore, the possible combinations range from only one to 32 processes (multi-threaded versions with 64 and 2 threads, respectively). The results of these and all the experiments in this section show the runtime of the total execution, i.e., not only the runtime of the alignment, but also of the additional steps such as loading the reference genome index, reading the sequences from the input file, printing the mappings into the intermediate files, and gathering the results into the output file.

The results indicate that the use of four or more processes per node is beneficial compared to applying only multi-threaded parallelism with one process and 64 threads. There are two reasons for this behavior: (1) the overhead due to synchronizing the accesses to the input file in the multi-threaded CUSHAW3 has a high impact for many threads; (2) the on-demand UPC++ distribution does not lead to workload imbalance when increasing the number of processes, and the only synchronization included in the UPC++ code is related to the read and update of one shared variable. Consequently, our UPC++ implementation not only allows to perform SRA on several nodes, but also can optimize the single-end alignments on single-node machines with many cores.

**Fig 5:**
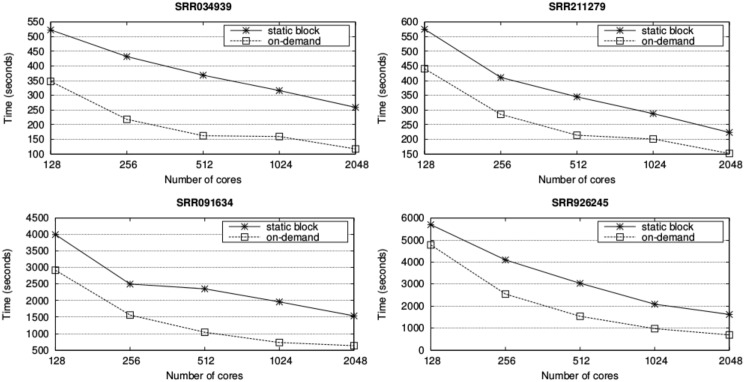
Runtime (seconds) of the single-end alignment varying the number of nodes. Results are shown for the UPC++ implementation with static and on-demand distributions.

Results for the single-end alignment of all the datasets using up to 32 nodes of the MOGON cluster are presented in Fig 5. Within each node, four processes with 16 threads each are used as this configuration provided the best performance in our previous single-node analysis. In order to test the impact of using our on-demand distribution, we have also included the runtime of a static variant of our UPC++ parallelization, which uses only one block per process. The results show that our on-demand distribution yields superior performance to the static distribution, which is often used by other tools such as pMap and pBWA, where the average improvement is 42.56% (50.35% for the largest experiments with 2,048 cores).

The scalability of our approach, compared to that of pMap [13], is presented in Table 2. As the current version of pMap does not provide support for CUSHAW3, we have adapted its source code to use CUSHAW3 and perform exactly the same alignment as our UPC++ implementation. In order to provide a fair comparison, all the experimental results for pMap are obtained with CUSHAW3 as underlying aligner. The pMap runtime was calculated by adding the times of the initial distribution, the alignment, and the final merging of the results. The baseline for the speedups is in all cases the runtime of the original multi-threaded CUSHAW3 tool using the 64 cores of one node. As expected, the speedups of our approach improve when increasing the size of the dataset, especially when increasing the lengths of the reads (sequences in the SRR926245 are longer than in other datasets). For instance, the runtime for aligning the smallest dataset (SRR034939) on 8 nodes is less than 3 minutes, which is difficult to improve using more computational resources due to the associated synchronization overhead. More efficient support by the UPC++ compiler to synchronize the accesses to the shared variable that indicates the next block of reads to analyze (e.g., semaphores, mutex, atomic functions) could help to improve these speedups. Nevertheless, our implementation obtains better scalability for problems with higher sequential runtime, as will be shown in the next subsection with the results for paired-end alignments. The results also show that our approach performs better than pMap in all cases. The expensive initial distribution and unbalanced workload of pMap limit the scalability of this framework (e.g., only a speedup of 2.94 can be achieved for SRR034939 using 32 nodes). Note that the UPC++ implementation sometimes obtains super-linear speedups for two and four nodes as it is executed with four processes per node, which is more efficient than the original CUSHAW3 version with 64 threads.

**Table 2:** Speedups of the UPC++ implementation and the pMap framework over CUSHAW3 with 64 threads for single-end alignment.

	UPC++					pMap				
Num. nodes →	2	4	8	16	32	2	4	8	16	32
SRR034939	2.77	4.41	5.93	6.03	8.21	1.73	2.19	2.73	3.02	2.94
SRR211279	2.30	3.55	4.73	4.86	6.67	1.81	1.99	2.46	3.63	3.49
SRR091634	2.09	3.91	5.86	8.35	9.61	1.66	2.56	3.39	4.73	5.69
SRR926245	2.09	3.92	6.50	10.24	14.44	1.79	3.09	5.03	7.11	8.84

We have not included results for pBWA [14] as it is based on BWA [32]. Nevertheless, according to the experimental results presented in [14], this tool never obtains parallel efficiency higher than 24% even for a small cluster with 240 cores. Thus, we can deduce that its scalability is supposed to be significantly lower than the results presented in Table 2.

### Paired-End Alignment

**Fig 6:**
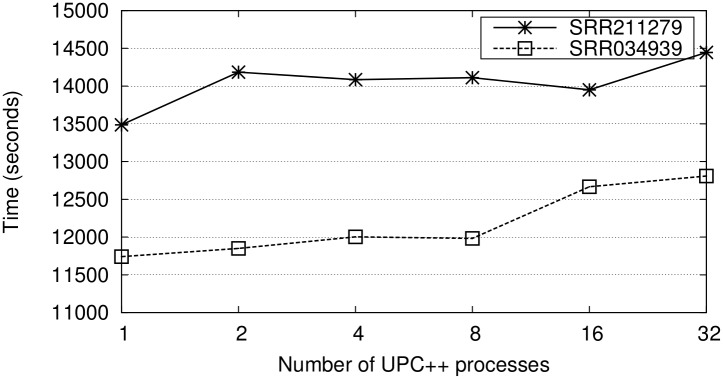
Runtime (seconds) of the paired-end alignment using the 64 cores of one node and varying the combinations of proccesses/threads (from one process with 64 threads to 32 processes with two threads each).

The analysis of the best combination of processes and threads on each node for paired-end alignment is presented in Fig 6. Note that the runtimes are in all cases more than 10 times higher than the single-end alignment because of the complexity of the algorithm. In this case the best approach consists in creating only one process per node and fully exploiting the multithreading capabilities of CUSHAW3, as the overhead due to thread synchronization to access the input file is not as significant as for single-end alignment. On one hand, as there are two input files, two threads can simultaneously access them without synchronization, which alleviates the overhead by a factor of two. On the other hand, the threads spend more time in the alignment of the pair of reads due to the complex calculations required and thus it is less common that several threads need to access the input files at the same time.

**Fig 7:**
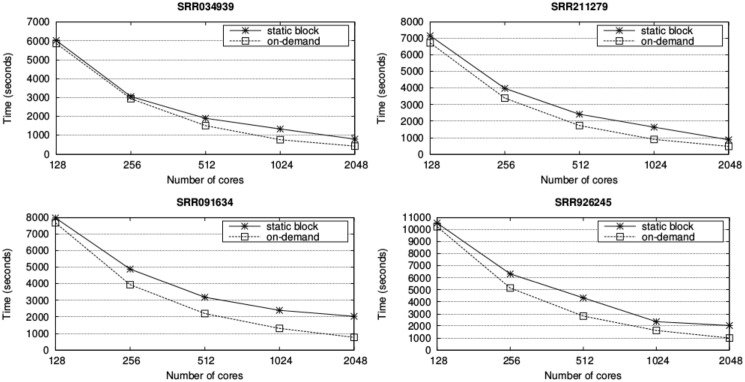
Runtime (seconds) of the paired-end alignment varying the number of nodes. Results are shown for the UPC++ implementation with static and on-demand distributions.

Fig 7 illustrates the results of the multi-node experiments for paired-end alignment. In this case, only one process per node with 64 threads is employed. Likewise, our approach is compared to an additional UPC++ version with a static block distribution. The degree of the improvement depends on the characteristics of the input dataset. For instance, the static distribution of the SRR091634 dataset with 32 nodes (2048 cores) creates blocks with exactly the same number of reads, but highly variable computational requirements. Consequently, the speedup of the on-demand version over the static distribution is as high as 2.65. Otherwise, the computational requirements of the blocks of the same distribution for the SRR211279 dataset are more balanced and thus the speedup is significantly lower (1.83) even for 32 nodes. On average, the speedup is 1.48 (2.09 for the experiments with 2048 cores).

Finally, the speedups over the CUSHAW3 paired-end alignment with 64 threads for our UPC++ implementation and pMap are given in Table 3. Scalability is better than for the single-end case (especially for the experiments with the highest amount of nodes) as also the sequential runtimes are higher. Another reason is that, as previously explained, less processes and threads require simultaneous access to the input files, thus improving the speedups. Regarding the comparison between pMap and the UPC++ version, the efficiency of the on-demand distribution depends on the dataset (if the blocks of pMap present balanced workload). In general, the benefit is higher than for the single-end experiments (on average, our UPC++ implementation is 1.99 times faster than pMap for 32 nodes).

**Table 3:** Speedups of the UPC++ implementation and the pMap framework over CUSHAW3 with 64 threads for paired-end alignment.

	UPC++					pMap				
Num. nodes →	2	4	8	16	32	2	4	8	16	32
SRR034939	2.00	3.99	7.76	15.29	27.28	1.76	3.38	6.35	9.85	16.29
SRR211279	2.00	3.97	7.77	15.04	28.25	1.89	3.60	6.55	11.18	17.13
SRR091634	1.98	3.85	6.90	11.64	19.83	1.69	2.96	5.16	5.37	6.44
SRR926245	1.94	3.87	7.08	12.27	19.99	1.85	3.43	6.03	9.71	12.80

## Conclusions

Recent advances of NGS technologies have established the need for fast tools to align sequence reads to reference genomes. However, to the best of our knowledge, most available tools to perform SRA on multi-core clusters are not able to efficiently exploit the computational capability of these systems. In this article we have therefore presented a parallel implementation of CUSHAW3 that obtains good scalability on multi-core clusters for accelerating SRA. Our approach provides the same high quality mappings as the original tool, but drastically reduces the runtime when executing on several nodes.

Our implementation is developed with UPC++ in order to take advantage of the strengths of the PGAS model (e.g., direct access to remote data or shared locks) and the object-oriented paradigm (e.g., inheritance or polymorphism). It is based on an on-demand approach that adapts the workload distribution according to the characteristics of the input dataset. We have evaluated our implementation using four Illumina datasets. Performance evaluations revealed that the on-demand approach always obtains better performance than a UPC++ counterpart with a static block distribution on a system with 32 nodes of 64 cores each, although its efficiency still depends on the characteristics of the input dataset. We have also tested that our implementation is, on average, 2.01 and 1.99 times faster than pMap using the 2048 cores for single-and paired-end alignments, respectively.

One additional advantage of the parallel approach described in this work is that it is flexible enough to be adapted to other short-read aligners, even to tools that exploit accelerators such as GPUs. Our UPC++ implementation does not modify the underlying method to align each read to the genome. Instead, it distributes the reads among processes and lets each process repeatedly call the method to align different reads. Thus, our implementation can also be regarded as a UPC++ framework which can be used to accelerate other seed-and-extend based aligners; i.e., users can replace the CUSHAW3 C++ method to align a read by their own approach. As future work, our plan is to include the UPC++ parallelization in GPU-based aligners such as CUSHAW2-GPU [33] so that they can exploit modern heterogeneous clusters and supercomputers with GPUs within the nodes. The source code of the parallel implementation described in this paper is publicly available at http://cushaw3.sourceforge.net.
